# Comparative genomic hybridization reveals population-based genetic alterations in hepatoblastomas

**DOI:** 10.1054/bjoc.2000.1390

**Published:** 2000-10

**Authors:** S G Gray, S Kytölä, T Matsunaga, C Larsson, T J Ekström

**Affiliations:** Laboratory for Molecular Development and Tumor Biology, Experimental Alcohol and Drug Addiction Research Section, Dept. of Clinical Neuroscience, Karolinska Institute, CMM, L8 01, Stockholm, S-171 76, Sweden; Endocrine Tumor Unit, Dept. of Molecular Medicine, Karolinska Hospital, CMM L8: 01, Stockholm, S-171 76, Sweden; Dept. of Pediatric Surgery, School of Medicine, Chiba University, 1-8-1 Inohana, Chuo-ku, Chiba, 260–0856, Japan

**Keywords:** hepatoblastoma, cytogenetics, Caucasian, Japanese, amplification

## Abstract

Hepatoblastoma is a malignant paediatric liver tumour. In order to approach the genetic background of this malignancy we have screened a panel of eighteen cases from Europe and Japan for chromosomal imbalances using comparative genomic hybridization (CGH). The most frequent losses included chromosomal regions 13q21–q22 (28%) and 9p22-pter (22%), while the most frequent gains occurred on 2q23–q24 (33%), 20q (28%) and 1q24–q25 (28%). A significant difference in CGH alterations between the tumours from patients of Caucasian and Japanese was revealed where loss of 13q was found only in the Japanese samples. In conclusion, the findings indicate several candidate regions for suppressor genes and oncogenes potentially involved in the hepatoblastomas of different ethnic origin. © 2000 Cancer Research Campaign


*British Journal of Cancer* (2000) **83**(8), 1020–1025
© 2000 Cancer Research Campaign
doi: 10.1054/ bjoc.2000.1390, available online at http://www.idealibrary.com on IDEAL®

### Comparative genomic hybridization reveals population-based genetic alterations in hepatoblastomas

**SG Gray[1]*, S Kytölä[2]*, T Matsunaga[3], C Larsson[2] and TJ Ekström[1]**

[1]Laboratory for Molecular Development and Tumor Biology, Experimental Alcohol and Drug Addiction Research Section, Dept. of Clinical Neuroscience, Karolinska Institute, CMM, L8 01, S-171 76 Stockholm, Sweden; [2]Endocrine Tumor Unit, Dept. of Molecular Medicine, Karolinska Hospital, CMM L8: 01, S-171 76 Stockholm, Sweden; [3]Dept. of Pediatric Surgery, School of Medicine, Chiba University 1-8-1 Inohana, Chuo-ku, Chiba, Japan 260–0856

**Summary** Hepatoblastoma is a malignant paediatric liver tumour. In order to approach the genetic background of this malignancy we have screened a panel of eighteen cases from Europe and Japan for chromosomal imbalances using comparative genomic hybridization (CGH). The most frequent losses included chromosomal regions 13q21–q22 (28%) and 9p22-pter (22%), while the most frequent gains occurred on 2q23–q24 (33%), 20q (28%) and 1q24–q25 (28%). A significant difference in CGH alterations between the tumours from patients of Caucasian and Japanese was revealed where loss of 13q was found only in the Japanese samples. In conclusion, the findings indicate several candidate regions for suppressor genes and oncogenes potentially involved in the hepatoblastomas of different ethnic origin. © 2000 Cancer Research Campaign

**Keywords:** hepatoblastoma; cytogenetics; Caucasian; Japanese; amplification

Hepatoblastoma is a malignant liver tumour most often found in infancy, with an incidence of 0.5–1.5 cases per million children (Perilongo and Shafford, 1999), although several cases of hepatoblastoma have also been reported for adults (Kuniyasu et al, 1996; Ahn et al, 1997; Parada et al, 1997). Whilst many cases of hepatoblastoma are sporadic, there are some familial inherited disorders of increased growth, most notably Beckwith–Wiedemann (BWS), for which there is a greatly increased chance of developing hepatoblastoma (Perilongo and Shafford, 1999). Congenital hepatoblastomas have also been reported, and are suggested to be a separate entity, distinct from hepatoblastomas which developed after neonatal age (Doss et al, 1998; Ammann et al, 1999).

The prognosis for affected children has improved dramatically following the introduction of preoperative chemotherapy with overall three year survival rates of 62–70% regardless of the modality used (Perilongo and Shafford, 1999). Several cytogenetic studies on hepatoblastomas have been described. Two cases of hepatoblastoma have been reported for children born with constitutional trisomy 18 (Bove et al, 1996; Teraguchi et al, 1997). Some of the most frequent constitutional changes observed in hepatoblastoma include: Trisomies 2, 7, 8, 19, and 20 (Mascarello et al, 1990; Tonk et al, 1994; Swarts et al, 1996; Sainati et al, 1998; Nagata et al, 1999; Park et al, 1999). The most frequent genetic abnormalities observed include loss of heterozygosity (LOH) in chromosomes 1 p and 11 p, and alterations to 2 q (Fletcher et al, 1991; Rodriguez et al, 1991; Anneren et al, 1992; Albrecht et al, 1994; Montagna et al, 1994; Kraus et al, 1996).

Less frequent cytogenetic changes have also been reported. These include translocations between the long arms of chromosomes 1 and 4 (Douglass et al, 1985; Schneider et al, 1997), a translocation between 1q and 14p (Park et al, 1999), insertion of 2q into 9p (Balogh et al, 1998) and others (reviewed in Nagata et al, 1999).

In an attempt to further define potential genetic regions of interest in hepatoblastomas, we have analysed a series of samples from two different ethnic backgrounds, Caucasian and Japanese, for numerical genomic imbalances using comparative genomic hybridization (CGH).

### MATERIALS AND METHODS

### Samples

A total of eighteen sporadic hepatoblastoma tumour biopsies were analysed (Table 1). Informed consent from the parents and approval for the study was granted by the local ethical committees. Eleven of the tumours were from patients of Caucasian origin while seven originated from Japan. The Caucasian samples were snap-frozen and stored at –70°C prior to analysis. The tumours were examined by standard histological examination and classified according to the systems used in the respective regions of origin (Morita et al, 1983; Weinberg and Finegold, 1983). The Caucasian samples were freeze-sectioned into 1 mm portions interrupted by 5 µm sections. Histopathological analysis was carried out on the 5 µm sections to obtain tissue profiles as described previously (Gray et al, 2000).

*Received 2 February 2000*
*Revised 7 June 2000*
*Accepted 15 June 2000*

*Correspondence to:* Dr TJ Ekström

*Contributed equally

**Table 1** Clinical data for the tumour samples used in this study

| Case No. | Ethnic origin | Age at dignosis (months) | Sex M/F | Histopathological subtype | Pre-operative chemotherapy |
|---|---|---|---|---|---|
| 1 | Caucasian | 6 | M | Epithelial | No |
| 2 | Caucasian | 19 | M | Epithelial | Yes |
| 3 | Caucasian | 19 | M | Epithelial | Yes |
| 4 | Caucasian | 22 | M | Epithelial/mesenchymal | Yes |
| 5 | Caucasian | 54 | M | Epithelial | Yes |
| 6 | Caucasian | 2 | M | Fetal | No |
| 7 | Caucasian | 12 | F | Fetal | Yes |
| 8 | Caucasian | 36 | M | N/A | Yes |
| 9 | Caucasian | 11 | F | Fetal | No |
| 10 | Caucasian | 13 | M | Fetal | No |
| 11 | Caucasian | 8 | F | Epithelial/mesenchymal | No |
| 12 | Japanese | 21 | M | Fetal | Yes |
| 13 | Japanese | 162 | F | Embryonal | Yes |
| 14 | Japanese | 19 | M | Embryonal | Yes |
| 15 | Japanese | 33 | F | Anaplastic | No |
| 16 | Japanese | 29 | M | Fetal | No |
| 17 | Japanese | 30 | M | Embryonal | Yes |
| 18 | Japanese | 23 | F | Fetal | Yes |

N/A – not available; M – Male; F – Female

### Comparative genomic hybridization

Genomic DNA was prepared by repeated phenol/chloroform extractions of tissue cells with 0.5% SDS and proteinase K (200 µg/ml final concentration). CGH was performed as previously described (Kallioniemi et al, 1992). Briefly, tumour DNA was labelled with FITC-dUTP (DuPont, Boston MA) by nick translation, and normal reference DNA was labeled with Texas Red (Vysis Inc., Downers Grove, IL, USA). Tumour and reference DNA were mixed with unlabelled Cot-1 DNA (Gibco, BRL), denatured, and applied onto slides with denatured metaphases of normal lymphocytes (Vysis Inc. Downers Grove, IL, USA). Using a commercial hybridization solution (Vysis Inc. Downers Grove, IL, USA), the slides were hybridized at 37°C for 48 h, and then were washed in 0.4 × SSC/0.3% NP-40 at 74°C for 2 mins and in 2 × SSC/0.1% NP-40 at room temperature for 1 min. After air drying, the slides were counterstained with DAPI (Vysis Inc. Downers Grove, IL, USA). Ten three-colour digital images (DAPI, FITC, and Texas Red fluorescence) were collected from the hybridizations using a Zeiss Axioplan 2 (Carl Zeiss Jena GmbH, Jena, Germany) epifluorescence microscope and Sensys (Photometrics) charge-coupled-device camera interfaced to a IPLab Spectrum 10 workstation (Signal Analytics Corporation, Vienna, VA, USA). Green-to-red ratios > 1.15 were considered as gains of genetic material, and ratios < 0.85 as losses. A green to red ratio > 1.5 was considered to be a high level amplification event. Heterochromatic regions, the short arm of the acrocentric chromosomes and chromosome Y were not included in the evaluation. Two control experiments were also performed. In the first, DNA from a normal male and DNA from a normal female were labelled and hybridized to normal male metaphases. For the second experiment DNA from a previously characterized breast cancer cell line (MPE600, Vysis Inc.) and DNA from a normal female were labelled and hybridized to normal male metaphases.

### Statistical analysis

Statistical analysis was carried out using StatView© (SAS Institute Inc., San Francisco, CA), and the analytical method used to examine the differences between genetic losses and gains based on ethnic background was the Fishers Exact Test.

### RESULTS

### Comparative genomic hybridization analysis

We used CGH to identify any chromosomal gains or losses in a series of sporadic hepatoblastomas (Table 1). The results are shown graphically in Figure 1, and have been tabulated for each individual tumour in Table 2. The minimal regions of losses for the most frequently altered chromosomes were 9p22-pter (22%), and 13q21–q22 (28%). The minimal regions of gain were 1q24–q25 (28%), 2q23–q24 (33%) and 20q (28%). In some of the samples showing gain of 2q, a distinct amplification of the region between 2q22–q24 was observed (Figure 2.) Four individuals were found to have no genetic losses or gains (Table 2).

One problem associated with the comparative genomic hybridization technique has been the problem of differential labelling of specific chromosomal regions. Such regions commonly include distal 1p, 22 and 16p (Larramendy et al, 1998). Our analysis of these samples revealed frequent losses involving chromosomes 1p (44%) and 16p (22%) using CGH. When we tested the samples for which we had matched normal liver from the same individual for loss of heterozygosity (LOH) at 1p, we were unable to demonstrate any LOH for the samples tested (data not shown). As such, any losses or gains involving this region, and for those of chromosomes 16p, 22 and the X chromosome were considered suspect, and discarded from the overall analysis. However, 1p LOH has been shown to occur in hepatoblastomas (Kraus et al, 1996), and thus the losses observed using CGH for these chromosomal regions, may represent submicroscopic or balanced genetic alterations in these samples.

© 2000 Cancer Research Campaign 

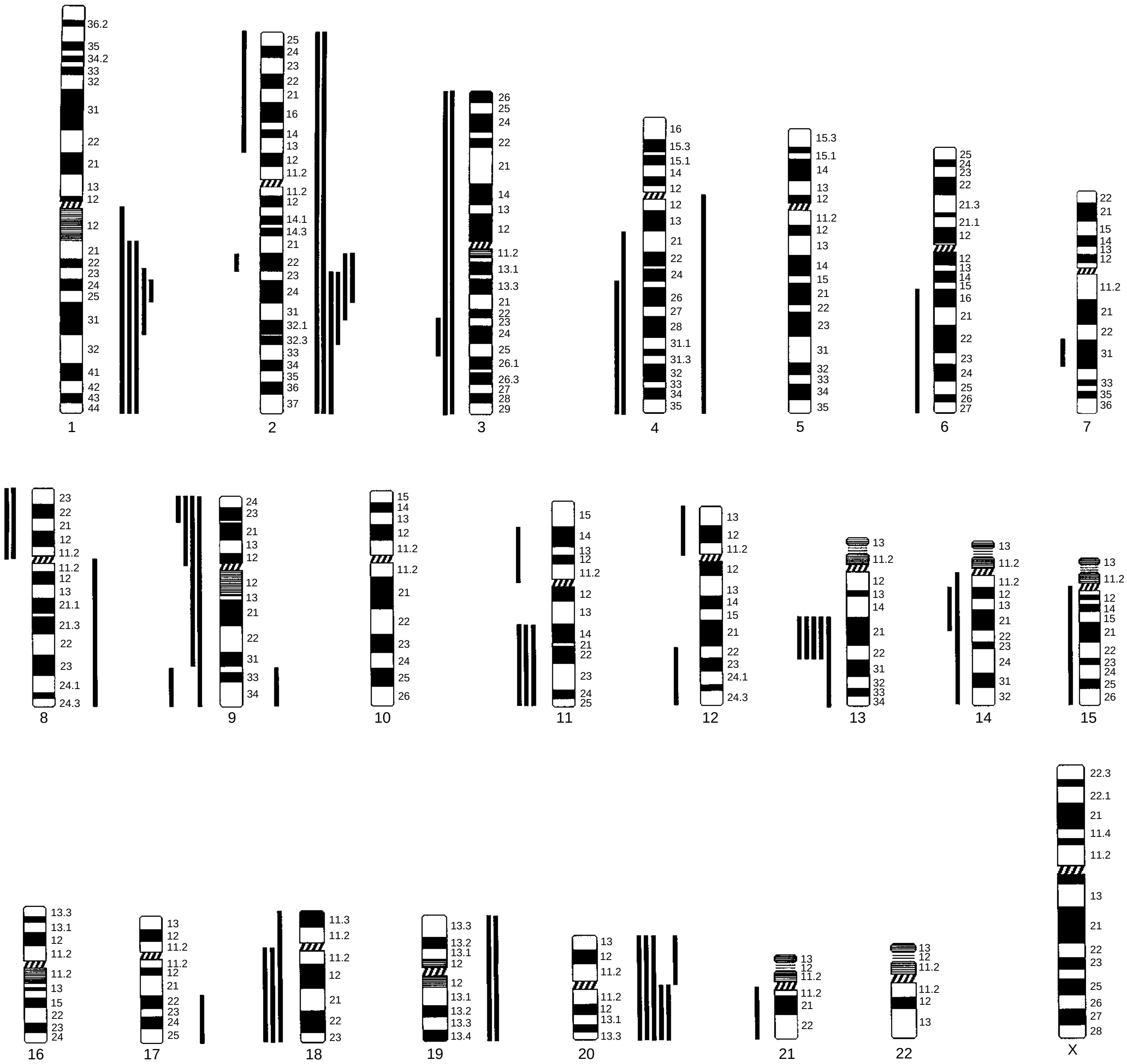

**Figure 1** DNA copy number changes detected by CGH in the hepatoblastoma samples. Each line represents one alteration detected in a tumour, with losses illustrated to the left and gains to the right of the ideograms

### Comparison of alterations in hepatoblastomas on the basis of ethnicity

We separated the genetic alterations in the primary tumours on the basis of ethnicity, to see if any significant patterns emerged. The results of this analysis are presented in Table 3. Several alterations occurred in the Japanese samples which were not present in the Caucasian samples. These were loss of 4q and 13q, and gain of chromosome 19. When we tested if any of these alterations were significant, only one, gain of 13q, was demonstrated to be statistically significant. The CGH profiles obtained for chromosome 13 in the Japanese samples are provided in Figure 3.

### DISCUSSION

Comparative genomic hybridization (CGH) was used to examine the samples for chromosomal losses and gains. Numerical imbalances were detected in the vast majority of cases (14/18). In the four cases without demonstratable chromosomal imbalances, submicroscopic or balanced genetic alterations cannot be excluded. In our samples, the most frequent losses found involved chromosomes 9 and 13. The most frequent gains occurred on chromosomes 1, 2 and 20. Other CGH analyses of hepatoblastomas have been reported recently. The results confirm the importance of chromosomes 1, 2, and 20 in hepatoblastoma (Steenman et al, 1999; Hu et al, 2000).

*© 2000 Cancer Research Campaign*

**Table 2** Losses and gains on the most frequently involved chromosome arms in relation to the total number of alterations in the tumor. The minimal regions involved are indicated

| Case No. | No of changes | 2q23–q24 gains | 20q gains | 1q24–q25 gains | 13q21–q22 losses | 9q22–pter losses | 18q losses | 11q14–qter losses | 3q23–q25 losses | Other gains | Other losses |
|---|---|---|---|---|---|---|---|---|---|---|---|
| 5 | 0 | | | | | | | | | | |
| 15 | 0 | | | | | | | | | | |
| 6 | 0 | | | | | | | | | | |
| 3 | 0 | | | | | | | | | | |
| 11 | 1 | 2q22–q24 | | | | | | | | | |
| 1 | 1 | | 20 | | | | | | | 4q | |
| 7 | 1 | | | | | | | | | | |
| 9 | 2 | 2q22–q31 | | 1q23–q31 | | | | | | | |
| 4 | 2 | 2 | | 1q21–qter | | | | | | | |
| 16 | 2 | | | | 13q21–qter | | | | | | 12p |
| 10 | 2 | | | 1q24–q25 | | | | | | | 8p |
| 17 | 3 | 2 | | | | | 18q | | | | 7q31 |
| 13 | 4 | | | | 13q21–q22 | | | 11q14–qter | | 9q32–qter, 19 | |
| 12 | 4 | | 20 | | 13q21–q22 | 9p22–qter | | | 3q23–q25 | | |
| 8 | 5 | 2q23–q32 | | | | | | | | 20 p | 2p13–pter, 9q32–qter, 12q22–qter |
| 18 | 7 | | 20q | | 13q21–q22 | 9p | | | | 8 q | 2q22, 4q25–qter, 8p |
| 2 | 8 | | 20 | 1q21–qter | | 9 | 18p | 11q14–qter | 3 | | 14, 15 q |
| 14 | 15 | 2q23–qter | 20q | 1q | 13q21–q22 | 9pter–q31 | 18 | 11q14–qter | 3 | 17q22–qter, 19 | 4q21–qter, 6q16–qter, 11c–p14, 14q12–q21, 21q |

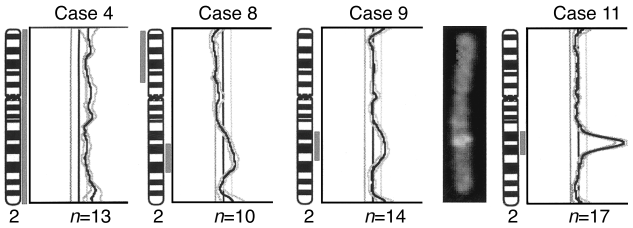

**Figure 2** Gains of chromosome 2 in hepatoblastomas. CGH ideograms and profiles for representative samples showing gains of chromosome 2 are presented. For Case 11, a distinct amplification of the region between 2q22-q24 was observed, and the digitized fluorescent image of chromosome 2 from this individual is also shown

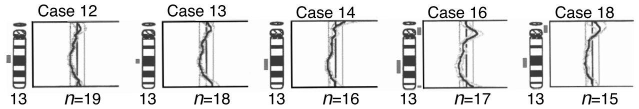

**Figure 3** Losses of chromosome 13 in hepatoblastomas. CGH ideograms and profiles for Japanese samples showing loss of chromosome 13 q presented

**Table 3** The most frequent CGH alterations in hepatoblastomas from Caucasian and Japanese cases

| | Caucasian | Japanese | *P* value[a] | Total |
|---|---|---|---|---|
| Gain of 2q23–q24 | 4/11 (36%) | 2/7 (29%) | n.s | 6/18 (33%) |
| Gain of 20 q | 2/11 (18%) | 3/7 (43%) | n.s | 5/18 (28%) |
| Gain of 1q24–q25 | 4/11 (36%) | 1/7 (14%) | n.s | 5/18 (28%) |
| Loss of 13q21–q22 | 0/11 (0%) | 5/7 (71%) | 0.025 | 5/18 (28%) |
| Loss of 9p22-pter | 1/11 (9%) | 3/7 (43%) | n.s | 4/18 (22%) |

[a]n.s = not significant

A hypothetical cytogenetic pattern of evolution for hepatoblastomas was proposed by Sainati and colleagues, for a subgroup of hepatoblastomas which suggested that the initial event was gain of chromosome 20, followed by gain of either chromosome 8 or chromosome 2 and leading to late gains of chromosomes 1 (1q21), 7 and 19 (Sainati et al, 1998). The results obtained in this study do not agree with this hypothetical pattern and suggest that multiple pathways may be involved with the development of hepatoblastoma.

A major difference between our analysis and the other CGH reports is that few genetic losses were observed in their samples, while many losses were observed in ours (Figure 1, Table 2). It is possible that many of the alterations observed may be due to the pre-operative chemotherapy (Table 1). However, individual Case 10 who did not receive any chemotherapy, has two chromosomal abnormalities as detected by CGH.

Very few alterations to 13q have been reported (Nagata et al, 1999). Our results however, demonstrate the importance of chromosome 13q in this tumour type. Five of our samples (28%) have a loss of all or part of chromosome 13q (Figure 3). The minimal region of loss in these samples is 13q21–q22. A review of the literature shows that the region encompassing 13q21 is frequently lost in 32 of 73 (47%) of all cancers examined using CGH (Knuutila et al, 1999). Thus, an important gene such as a tumour suppressor may lie within this chromosomal region.

© 2000 Cancer Research Campaign

One individual, Case 11, has only one genetic alteration which involves gain of 2q22–q24 (Table 2). The gain of 2q may play a significant role in hepatoblastoma development, since in addition to this individual, several others were found with a gain of 2q22–q32 (Table 2, Figure 2). Several other papers have shown trisomy 2 and partial trisomy of 2q in hepatoblastoma (Nagata et al, 1999). More significantly, structural abnormalities involving 2q have also been reported involving the region of 2q (2q23–q32), which we have observed to be amplified (Nagata et al, 1999) indicating that this region may be very important in the development of hepatoblastomas. A recent analysis of hepatoblastomas using CGH by Perlman and colleagues has also demonstrated a high level amplification of this region (Hu et al, 2000). One investigation examining the amplification of several well characterized oncogenes such as N-MYC was unable to find any amplification in hepatoblastomas (Mares et al, 1998). If this amplification is due to the presence of a potential oncogene, a possible candidate gene may be the fibroblast activation protein alpha (FAP) which has been mapped to 2q23 (Mathew et al, 1995). This is a cell surface antigen shown to be selectively expressed in several cancers, but has also been shown to be transiently expressed in fetal mesenchymal tissues (Scanlan et al, 1994). The FAP protein is a surface bound serine protease and has been proposed to play a role in the invasion of cancer cells into the extracellular matrix (Chen, 1996).

Another potential candidate may be a multiple drug resistance (MDR) gene. Recently, a CGH study examining the genetic changes in human ovarian carcinoma cell lines resistant to cisplatin found that gain of region 2q14.1–q33 occurred in 5 of 6 cell lines examined (Wasenius et al, 1997). The gene for *MDR*1 encodes a p-glycoprotein and belongs to the ATP-binding cassette (ABC) transporter. A novel liver specific ABC transporter *BSEP*, was recently identified and maps to 2q24, and is involved in a rare childhood liver disorder, in progressive familial intrahepatic cholestasis (PFIC) (Strautnieks et al, 1998). Thus, the frequent alterations to chromosome 2 frequently observed in hepatoblastomas, involving trisomies, amplifications, and translocations, may relate to drug resistance being engendered by the preoperative treatment of the individuals.

However, two of the individuals who did not receive any chemotherapy prior to surgery also have gains within this region (Table 1, Table 2), and therefore, it seems more likely, that if the candidate gene *BSEP* is involved in hepatoblastoma development, it may be through a defect in its function as the major canicular bile salt export pump. Gain of 2q22–q24 in hepatoblastomas may be of critical importance in the development or pathogenesis of this tumour. Because chromosome 2q is so frequently altered in hepatoblastomas, a concerted effort should be undertaken to determine the gene involved.

Finally, we have demonstrated an ethnographical difference in hepatoblastomas. Loss of 13q only occurs in the Japanese samples. The significance of this is currently unknown. One possibility may be hepatitis infection, although this is not common in Japan. Hepatocellular carcinoma (HCC) is frequently associated with hepatitis infections. Several studies-have demonstrated that loss of chromosome 13q is frequent in HCCs (ranging between 20–32.4%) (Nagai et al, 1997; Lin et al, Sakakura et al, 1999; Sheu et al, 1999). However, there has been no correlation between loss of 13q and hepatitis, and in two studies, loss of 16q appears to be the most significant genetic abnormality associated with hepatitis infected HCC (Lin et al, 1999; Sheu et al, 1999). The samples from Japan used in our study were all hepatitis B antigen (HBs-Ag) negative, indicating that hepatitis B is not a factor. Some of the samples have also been tested for hepatitis C (HCV). Of the ones tested, only sample 12 was positive for HCV. The other samples tested for HCV (samples 17–19) were negative. Thus, the loss of 13q may not be related to hepatitis C infection as one of the samples tested (sample 18) was HCV negative while one (sample 12) was HCV positive.

Clearly, a much larger study using CGH should be undertaken for hepatoblastomas to define more clearly the genetic alterations in hepatoblastoma for both general and demographic or regional alterations.

### ACKNOWLEDGEMENTS

This study was supported by grants from the Swedish Cancer Foundation (TJE, CL), the Childrens Cancer Foundation of Sweden (TJE), and the Torsten and Ragnar Söderberg Foundations (CL). The authors would like to thank Bengt Sandstedt for his help with the histopathological examinations.

### REFERENCES

Ahn HJ, Kwon KW, Choi YJ, Kim HJ, Hong SP, Oh D and Chung JS (1997) Mixed hepatoblastoma in an adult – a case report and literature review. *Journal of Korean Medical Science* **12**: 369–373

Albrecht S, von Schweinitz D, Waha A, Kraus JA, von Deimling A and Pietsch T (1994) Loss of maternal alleles on chromosome arm 11p in hepatoblastoma. *Cancer Research* **54**: 5041–5044

Ammann RA, Plaschkes J and Leibundgut K (1999) Congenital hepatoblastoma: a distinct entity? *Medical and Pediatric Oncology* **32**: 466–468

Anneren G, Nordlinder H and Hedborg F (1992) Chromosome aberrations in an alpha-fetoprotein-producing hepatoblastoma. *Genes, Chromosomes and Cancer* **4**: 99–100

Balogh E, Swanton S, Kiss C, Jakab ZS, Secker-Walker LM and Oláh É (1998) Fluorescence in situ hybridization reveals trisomy 2q by insertion into 9p in hepatoblastoma. *Cancer Genetics and Cytogenetics* **102**: 148–150

Bove KE, Soukup S, Ballard ET and Ryckman F (1996) Hepatoblastoma in a child with trisomy 18: cytogenetics, liver anomalies, and literature review. *Pediatric Pathology and Laboratory Medicine* **16**: 253–262

Chen WT (1996) Proteases associated with invadopodia, and their role in degradation of extracellular matrix. *Enzyme and Protein* **49**: 59–71

Doss BJ, Vicari J, Jacques SM and Qureshi F (1998) Placental involvement in congenital hepatoblastoma. *Pediatric and Developmental Pathology* **1**: 538–542

Douglass EC, Green AA, Hayes FA, Etcubanas E, Horowitz M and Wilimas JA (1985) Chromosome 1 abnormalities: a common feature of pediatric solid tumors. *Journal of the National Cancer Institute* **75**: 51–54

Fletcher JA, Kozakewich HP, Pavelka K, Grier HE, Shamberger RC, Korf B and Morton CC (1991) Consistent cytogenetic aberrations in hepatoblastoma: a common pathway of genetic alterations in embryonal liver and skeletal muscle malignancies? *Genes, Chromosomes and Cancer* **3**: 37–43

Gray SG, Eriksson T, Ekström C, von Schweinitz D, Kogner P, Sandstedt B, Pietsch T and Ekström TJ (2000) Altered expression of members of the IGF-axis in hepatoblastomas. *British Journal of Cancer* **82**(9): 1561–1567

Hu J, Wills M, Baker BA and Perlman EJ (2000) Comparative genomic hybridization analysis of hepatoblastomas. *Genes, Chromosomes and Cancer* **27**: 196–201

Kallioniemi A, Kallioniemi OP, Sudar D, Rutovitz D, Gray JW, Waldman F and Pinkel D (1992) Comparative genomic hybridization for molecular cytogenetic analysis of solid tumors. *Science* **258**: 818–821

Knuutila S, Aalto Y, Autio K, Bjorkqvist AM, El-Rifai W, Hemmer S, Huhta T, Kettunen E, Kiuru-Kuhlefelt S, Larramendy ML, Lushnikova T, Monni O, Pere H, Tapper J, Tarkkanen M, Varis A, Wasenius VM, Wolf M and Zhu Y (1999) DNA copy number losses in human neoplasms. *American Journal of Pathology* **155**: 683–694

Kraus JA, Albrecht S, Wiestler OD, von Schweinitz D and Pietsch T (1996) Loss of heterozygosity on chromosome 1 in human hepatoblastoma. *International Journal of Cancer* **67**: 467–471

*© 2000 Cancer Research Campaign*

Kuniyasu H, Yasui W, Shimamoto F, Fujii K, Nakahara M, Asahara T, Dohi K and Tahara E (1996) Hepatoblastoma in an adult associated with c-met proto-oncogene imbalance. *Pathology International* **46**: 1005–1010

Larramendy ML, El-Rafai W and Knuutila S (1998) Comparison of fluorescein isothiocyanate- and Texas red-conjugated nucleotides for direct labeling in Comparative Genomic Hybridization. *Cytometry* **31**: 174–179

Lin YW, Sheu JC, Huang GT, Lee HS, Chen CH, Wang JT, Lee PH and Lu FJ (1999) Chromosomal abnormality in hepatocellular carcinoma by comparative genomic hybridisation in Taiwan. *European Journal of Cancer* **35** 652–658

Mares J, Polanska V, Gorgens H, Sedlacek Z, Marikova T, Bocek P, Kodet R, Schackert J and Goetz P (1998) Oncogene amplification and expression in pediatric solid tumors. *Neoplasma* **45**: 123–127

Mascarello JT, Jones MC, Kadota RP and Krous HF (1990) Hepatoblastoma characterized by trisomy 20 and double minutes. *Cancer Genetics and Cytogenetics* **47**: 243–247

Mathew S, Scanlan MJ, Mohan Raj BK, Murty VV, Garin-Chesa P, Old LJ, Rettig WJ and Chaganti RS (1995) The gene for fibroblast activation protein alpha (FAP), a putative cell surface-bound serine protease expressed in cancer stroma and wound healing, maps to chromosome band 2q23. *Genomics* **25**: 335–337

Montagna M, Menin C, Chieco-Bianchi L and D'Andrea E (1994) Occasional loss of constitutive heterozygosity at 11p15.5 and imprinting relaxation of the IGF-II maternal allele in hepatoblastoma. *Journal of Cancer Research and Clinical Oncology* **120**: 732–736

Morita K, Okabe I, Uchino J, Watanabe I, Iwabuchi M, Matsuyama S, Takahashi H, Nakajo T, Hirai Y, Tsuchida Y, Katsumata K, Hasegawa, H, Nishi T, Okamoto E and Ikeda K (1983) The proposed Japanese TNM classification of primary liver carcinoma in infants and children. *Japanese Journal of Clinical Oncology* **13**: 361–369

Nagai H, Pineau P, Tiollais P, Buendia MA and Dejean A (1997) Comprehensive allelotyping of human hepatocellular carcinoma. *Oncogene* **14**: 2927–2933

Nagata T, Mugishima H, Shichino H, Suzuki T, Chin M, Koshinaga S, Inoue M and Harada K (1999) Karyotypic analyses of hepatoblastoma – report of two cases and review of the literature suggesting chromosomal loci responsible for the pathogenesis of this disease. *Cancer Genetics and Cytogenetics* **114**: 42–50

Parada LA, Bardi G, Hallen M, Hagerstrand I, Tranberg KG, Mitelman F and Johansson B (1997) Cytogenetic abnormalities and clonal evolution in an adult hepatoblastoma. *American Journal of Surgical Pathology* **21**: 1381–1386

Park JP, Ornvold KT, Brown AM and Mohandas TK (1999) Trisomy 2 and 19, and tetrasomy 1q and 14 in hepatoblastoma. *Cancer Genetics and Cytogenetics* **115**: 86–87

Perilongo G and Shafford EA (1999) Liver tumours. *European Journal of Cancer* **35**: 953–958

Rodriguez E, Reuter VE, Mies C, Bosl GJ and Chaganti RS (1991) Abnormalities of 2q: a common genetic link between rhabdomyosarcoma and hepatoblastoma? *Genes, Chromosomes and Cancer* **3**: 122–127

Sainati L, Leszl A, Stella M, Montaldi A, Perilongo G, Rugge M, Bolcato S, Iolascon A and Basso G (1998) Cytogenetic analysis of hepatoblastoma: hypothesis of cytogenetic evolution in such tumors and results of a multicentric study. *Cancer Genetics and Cytogenetics* **104**: 39–44

Sakakura C, Hagiwara A, Taniguchi H, Yamaguchi T, Yamagishi H, Takahashi T, Koyama K, Nakamura Y, Abe T and Inazawa J (1999) Chromosomal aberrations in human hepatocellular carcinomas associated with hepatitis C virus infection detected by comparative genomic hybridization. *British Journal of Cancer* **80**: 2034–2039

Scanlan MJ, Raj BK, Calvo B, Garin-Chesa P, Sanz-Moncasi MP, Healey JH, Old LJ and Rettig WJ (1994) Molecular cloning of fibroblast activation protein alpha, a member of the serine protease family selectively expressed in stromal fibroblasts of epithelial cancers. *Proceedings of the National Academy of Sciences of the United States of America* **91**: 5657–5661

Schneider NR, Cooley LD, Finegold MJ, Douglass EC and Tomlinson GE (1997) The first recurring chromosome translocation in hepatoblastoma: der(4)t(1;4)(q12;q34). *Genes, Chromosomes and Cancer* **19**: 291–294

Sheu JC, Lin YW, Chou HC, Huang GT, Lee HS, Lin YH, Huang SY, Chen CH, Wang JT, Lee PH, Lin JT, Lu FJ and Chen DS (1999). Loss of heterozygosity and microsatellite instability in hepatocellular carcinoma in Taiwan. *British Journal of Cancer* **80**: 468–476

Steenman M, Tomlinson G, Westerveld A and Mannens M (1999) Comparative genomic hybridization analysis of hepatoblastomas: additional evidence for a genetic link with Wilms tumor and rhabdomyosarcoma. *Cytogenetics and Cell Genetics* **86**: 157–161

Strautnieks SS, Bull LN, Knisely AS, Kocoshis SA, Dahl N, Arnell H, Sokal E, Dahan K, Childs S, Ling V, Tanner MS, Kagalwalla AF, Nemeth A, Pawlowska J, Baker A, Mieli-Vergani G, Freimer NB, Gardiner RM and Thompson RJ (1998) A gene encoding a liver-specific ABC transporter is mutated in progressive familial intrahepatic cholestasis. *Nature Genetics* **20**: 233–238

Swarts S, Wisecarver J and Bridge JA (1996) Significance of extra copies of chromosome 20 and the long arm of chromosome 2 in hepatoblastoma. *Cancer Genetics and Cytogenetics* **91**: 65–67

Teraguchi M, Nogi S, Ikemoto Y, Ogino H, Kohdera U, Sakaida N, Okamura A, Hamada Y and Kobayashi Y (1997) Multiple hepatoblastomas associated with trisomy 18 in a 3-year-old girl. *Pediatric Hematology and Oncology* **14**: 463–467

Tonk VS, Wilson KS, Timmons CF and Schneider NR (1994) Trisomy 2, trisomy 20, and del(17p) as sole chromosomal abnormalities in three cases of hepatoblastoma. *Genes, Chromosomes and Cancer* **11**: 199–202

Wasenius VM, Jekunen A, Monni O, Joensuu H, Aebi S, Howell SB and Knuutila S (1997) Comparative genomic hybridization analysis of chromosomal changes occurring during development of acquired resistance to cisplatin in human ovarian carcinoma cells. *Genes, Chromosomes and Cancer* **18**: 286–291

Weinberg AG and Finegold MJ (1983) Primary hepatic tumors of childhood. *Human Pathology* **14**: 512–537

*© 2000 Cancer Research Campaign* 
